# *Yersiniapseudotuberculosis* serotype O:1 infection in a captive Seba’s short tailed-fruit bat (*Carollia perspicillata)* colony in Switzerland

**DOI:** 10.1186/s12917-021-02796-y

**Published:** 2021-02-27

**Authors:** K. Hahn, I. B. Veiga, M. Schediwy, D. Wiederkehr, M. Meniri, M. Schneeberger, P. Rüegg-van den Broek, C. Gurtner, N. J. Fasel, S. Kittl, M. Fredriksson-Ahomaa, S. Schmitt, N. Stokar-Regenscheit

**Affiliations:** 1 Vetsuisse Faculty, Institute of Animal Pathology, University of Bern, Bern, Switzerland; 2 Institute of Virology and Immunology, Bern, Switzerland; 3 Vetmedics Praxis Dr. Schediwy GmbH, Muri-Gümligen, Switzerland; 4 Department of Agronomy, School of Agricultural, Forest and Food Sciences HAFL, Bern University of Applied Sciences, Zollikofen, Switzerland; 5 Institute of Biology, University of Neuchâtel, Neuchâtel, Switzerland; 6 Vetsuisse Faculty, Institute for Food Safety and Hygiene, Section of Veterinary Bacteriology, University of Zurich, Zurich, Switzerland; 7 Foundation Papiliorama, Kerzers, Switzerland; 8 Department of Ecology and Evolution, University of Lausanne, Lausanne, Switzerland; 9 Vetsuisse Faculty, Institute of Veterinary Bacteriology, University of Bern, Bern, Switzerland; 10 Department of Food Hygiene and Environmental Health, Faculty of Veterinary Medicine, University of Helsinki, Helsinki, Finland

## Abstract

**Background:**

Between February and April 2016, a slight increase in mortality was observed in a colony consisting of 400 captive Seba’s short-tailed bats (*Carollia perspicillata*). These animals cohabited with other nocturnal animal species in a dome of a private zoo in Switzerland.

**Results:**

Gross and histological analysis of two (14.3%) out of the 13 animals submitted for necropsy within this period revealed a necrosuppurative pneumonia, hepatitis, splenitis, enterocolitis, and endometritis, with abundant intralesional colonies of Gram-negative rods. *Yersinia* (*Y.*) *pseudotuberculosis* serotype O:1 and biotype 1 belonging to the sequence type ST90 was isolated from the affected organs in both animals. Following this diagnosis, ¼ of the colony (99 animals) was culled and submitted for gross and histopathological analysis, and a bacterial culture selective for *Yersinia* spp. of lung, liver, and spleen was performed. From these 99 animals, one gravid female was tested and found to be positive for *Y. pseudotuberculosis* in the absence of clinical symptoms and histopathological lesions*.* PCR analysis of altogether three bacterial isolates for virulence factors revealed the presence of the *ail* gene, and one isolate was also positive for the *virF* and *yadA* plasmid genes.

**Conclusions:**

These findings suggest that *Carollia perspicillata* are susceptible to lethal yersiniosis but do not represent a regular reservoir for *Y. pseudotuberculosis*. Culling of ¼ of the population was sufficient to limit the spread of this infection among the colony. Moreover, no infections were detected in cohabitant nocturnal animals and caretakers, indicating that the zoonotic risk in this case was low.

## Background

*Yersinia (Y.) pseudotuberculosis* is a zoonotic Gram-negative rod with a global distribution, which can cause disease in a broad range of mammalian and avian species [1]. This organism is transmitted faecal-orally, and ingestion of contaminated food or water is considered the major source of infection in humans and animals. Rodents and birds are considered to be reservoir hosts and display a milder or even asymptomatic course of infection [2]. Severe infection associated with high mortality has been described not only in hares during epizootics, but also in several zoo animals, including monkeys and larger rodents such as pacas and capybaras [2–4].

Bats act as important reservoirs for several bacterial, viral, protozoal and fungal pathogens, and their potential role in the transmission of zoonoses is receiving growing attention [5]. Following detection of *Y. pseudotuberculosis* in free-living bats (*Myotis myotis*) in Germany, a potential role of these animals as *Yersinia* spp. reservoirs is discussed [6, 7]. Moreover, two *Y. pseudotuberculosis* outbreaks in captive bat colonies of Egyptian rousette bats (*Rousettus aegyptiacus)* were associated with high morbidity and mortality rates [1, 8].

*Carollia (C.) perspicillata* is a common species naturally occurring in the Neotropics that belongs to the *Phyllostomidae* family. It is a medium sized bat, with a wingspan of about 30 cm for an adult, weight of approximately 20 g. The average lifespan in captivity is 12 years [9, 10]. Females reach sexual maturity at approximately 1 year of age and males become mature between 1 and 2 years [9]. They can have two litters per year in close synchrony with fruit availability [11].

The current report describes the detection of *Y. pseudotuberculosis* serotype O:1 infection in three out of 112 animals in an indoor-housed colony of captive *C. perspicillata* in a private zoo in Switzerland.

## Results

### *Y. pseudotuberculosis* infection is not associated with a statistically significant increase in mortality within *C. perspicillata* colony

**Fig. 1:**
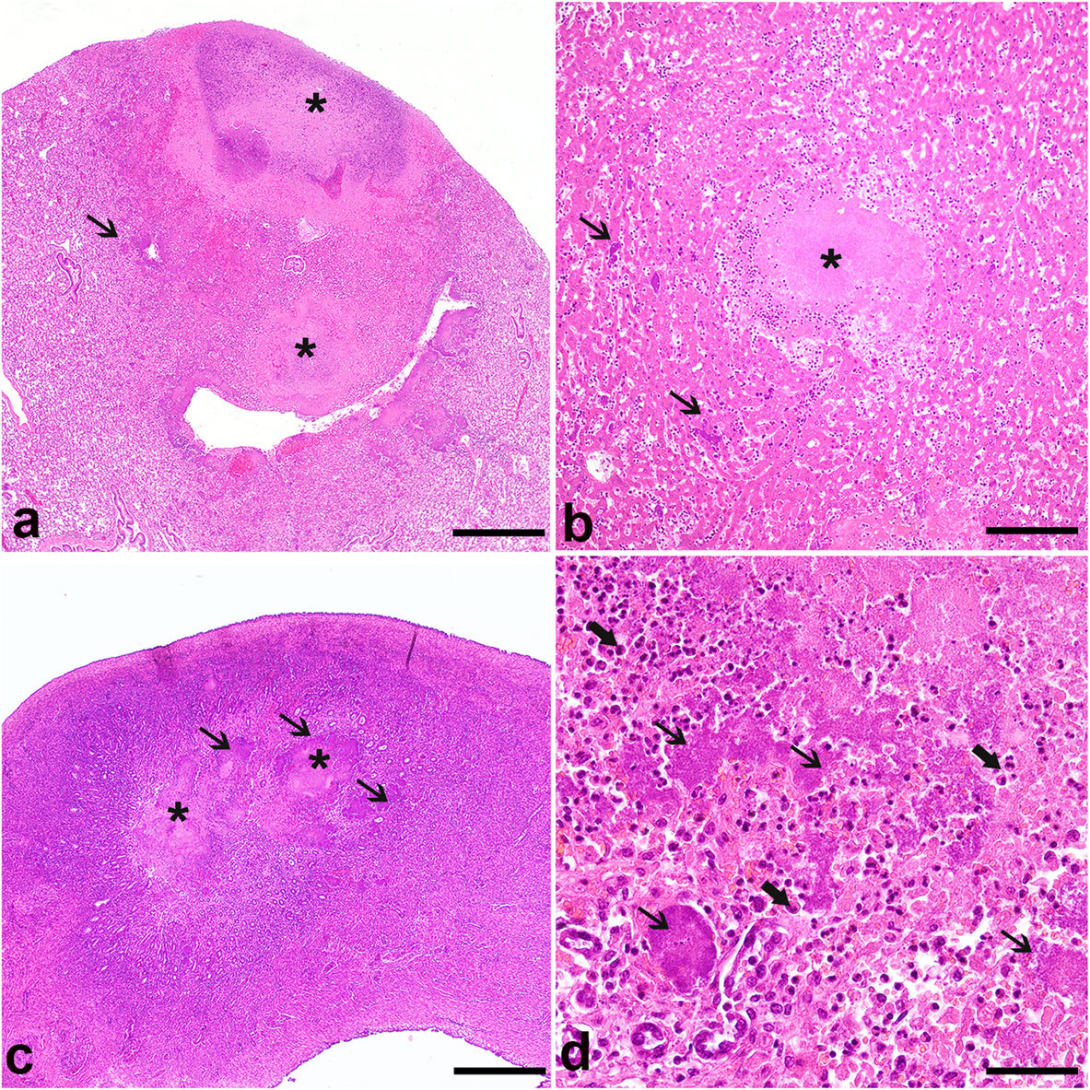
Necrosuppurative lesions identified histologically in the lung (**a**), liver (**b**), and uterus (**c** and **d**) from ID1**.** These were characterized by central necrotic areas (asterisks) surrounded by abundant intralesional colonies of bacterial rods (thin arrows) and variable numbers of degenerated neutrophils (thick arrows). HE, Bar 500 μm (**a** and **c**), 200 μm (**b**) and 50 μm (**d**), respectively

A slight mortality increase among bats of this species was observed between February and April 2016 during a birth peak. Thirteen bats overall were submitted for necropsy. Some of these animals had been found dead and frozen shortly prior to February 2016. The majority of the necropsied bats were female (8.6%) and/or young animals (61.5%) (Table 1). Of these animals, one adult female was known to have aborted about a week prior to death (ID1). The other animal was an adult male (ID2) that displayed multifocal white foci in the liver measuring approximately 1–2 μm in diameter at necropsy, while the lungs were reddened and of increased consistency. Histopathological examination of both animals revealed a severe, multifocal to coalescing, acute, necrosuppurative hepatitis and pneumonia, with abundant intralesional bacterial rod colonies (Fig. 1a and b) that stained red in the Gram staining. Additional histopathological lesions included a severe necrosuppurative enterocolitis, lymphadenitis, splenitis, stomatitis and glossitis, as well as an endometritis in the female (Fig. 1c and d). In both animals (1,4; 3% of the examined animals)*,* a high load of *Y. pseudotuberculosis* was cultivated from the liver, and a low bacterial load was isolated from the lungs and kidneys. In three of the remaining examined animals, a mild to moderate interstitial pneumonia (ID10, 12 and 13; 23% of the examined animals) was present. Three young bats were diagnosed with foetal atelectasis with intra-alveolar deposition of keratin scales (ID4, 6 and 7; 23% of the examined animals). One animal displayed multifocal acute haemorrhages in the musculature, representing trauma-associated lesions (ID6; 7.7% of the examined animals). The five remaining animals were inconspicuous both in the gross and histopathological analysis (ID 3, 5, 8, 9 and 11; 38.4% of the examined animals) (Table 1). Between January 2014 and April 2016, the average number of dead bats in this colony was 3.25 per month (min:0, max: 9), and the highest mortality rate registered was observed in July 2014 (*n* = 9, coefficient: 5.730, t-value = 3.627) (Table 2). The number of bats found dead at the time that *Y. pseudotuberculosis* was diagnosed within this colony did not differ significantly from the months measured before.

**Table 1:** Characterization of *C. perspicillata* submitted to pathological and bacteriological examination between February and April 2016

ID	Sex	Age group	Gross and histological findings	Bacteriological analysis
**1**	f	adult	Necrosuppurative metritis, pneumonia, hepatitis and splenitis	*Y. pseudotuberculosis* positive (liver +++, lung +, kidney +)
**2**	m	adult	Necrosupppurative pneumonia, hepatitis, enterocolitis, splenitis, lymphadenitis, glossitis and stomatitis	*Y. pseudotuberculosis* positive (liver +++, lung +, kidney +)
**3**	f	adult	Unremarkable	negative
**4**	f	young	Fetal atelectasis with intraalveolar keratin scales	negative
**5**	unknown	newborn	Unremarkable	negative
**6**	f	young	1) Multifocal acute hemorrhages in the musculature 2) Fetal atelectasis with intraalveolar keratin scales	negative
**7**	f	young	Fetal atelectasis with intraalveolar keratin scales	negative
**8**	f	young	Unremarkable	negative
**9**	f	young	Unremarkable	negative
**10**	f	young	Mild interstitial pneumonia	negative
**11**	f	young	Unremarkable so far as assessable due to severe autolysis	negative
**12**	f	young	Moderate interstitial pneumonia	negative
**13**	f	unknown	Moderate interstitial pneumonia	negative

**Table 2:** Mortality rate between January 2014 and April 2016 in *C. perspicillata* colony

Month	Newborn	Young	Adult	Unknown	Total	Coefficient	t-value
Jan 14		2	1		3	−0.192	− 0.122
Feb 14		1	1		2	− 1.446	− 0.916
Mar 14		4	4		8	4.907	3.106
Apr 14		1			1	−2.358	−1.493
May 14		1			1	−2.137	−1.353
Jun 14	1	1		1	3	−0.402	−0.255
Jul 14	2	2	2	3	9	5.73	**3.627**
Aug 14		2	2		4	0.465	0.295
Sep 14			4		4	0.642	0.406
Oct 14	1	1	2	1	5	1.73	1.095
Nov 14		1	2		3	−0.314	−0.199
Dec 14		1	2		3	−0.137	−0.087
Jan 15	1				1	−2.137	−1.353
Feb 15		1			1	−2.182	−1.381
Mar 15		2	2		4	0.907	0.574
Apr 15			1	1	2	−1.137	−0.72
May 15					0	−3.182	−2.014
Jun 15		1	2		3	−0.049	− 0.031
Jul 15			2		2	−1.137	−0.72
Aug 15			1		1	−2.137	−1.353
Sep 15		1	1		2	−1.137	−0.72
Oct 15			3		3	−0.358	−0.227
Nov 15		2	1	4	7	3.818	2.417
Dec 15			2		2	−1.491	−0.943
Jan 16	1		3	1	5	1.686	1.067
Feb 16		1	5		6	2.642	1.672
Mar 16		3	1		4	0.686	0.434
Apr 16	1	1			2	−1.281	−0.81

The coefficients and t-values of the outliers’ detection analysis was performed only on the total number of dead bats. The critical value was set at 3.5 and significant t-value (July 2014) is in bold. This finding was attributed to an ongoing birth peak in combination with increased stress due to an unusually high number of visitors

### Low prevalence of *Y. pseudotuberculosis* infection in the *C. perspicillata* colony

Following *Y. pseudotuberculosis* detection within the colony, approximately ¼ of the *C. perspicillata* population was culled in June 2016. Out of the 99 clinically healthy animals that were captured and culled, *Y. pseudotuberculosis* was detected in a pooled organ sample comprising liver, lung and spleen from one clinically healthy female gravid bat in the absence of gross and histopathological lesions. Also, no signs of inflammation were detected macroscopically and histologically in any of the remaining 98 bats.

### *Y. pseudotuberculosis* isolates from *C. perspicillata are* characterized as serotype O:1 and biotype 1

All three *Y. pseudotuberculosis* obtained isolates (16–1261/1, 16–1261/2 and 16–457/29) were characterized as serotype O:1 and biotype 1 following slide agglutination. They all belonged to the sequence type ST90 and harboured the chromosomal *ail* gene. In addition, the isolate 16–457/29 from the clinically healthy gravid female culled during depopulation was positive for the plasmid genes *virF* and *yadA* (Table 3).

**Table 3:** Characterization of *Y. pseudotuberculosis* isolates obtained in the bacteriological analysis from the three infected *C. perspicillata*

Isolate	16–1261/1	16–1261/2	16–457/29
	(ID1)	(ID2)	
**Serotype**	O:1	O:1	O:1
**Biotype**	1	1	1
**raffinose**	–	–	–
**melibiose**	+	+	+
**citrate**	–	–	–
***virF***	–	–	+
***yadA***	–	–	+
***ail***	+	+	+
**Sequence type**	ST90	ST90	ST90

### No indication of Y. pseudotuberculosis infection in cohabitant nocturnal animal species and caretakers

None of the nocturnal animals that inhabited the same dome as the *C. perspicillata* were sick, died or were euthanized between February and April 2016.

Moreover, *Yersinia* species were not diagnosed in any of the zoo animals submitted for necropsy and in the pooled faecal samples submitted for routine bacteriological analysis since April 2016 up to today. Also, no cases of *Yersinia* infection were detected among the animal caretakers.

## Discussion

Close observation of an indoor-housed captive colony of *C. perspicillata* housed in a dome from a private zoo in Switzerland led to the detection of *Y. pseudotuberculosis* infection between February and April 2016. The infection occurred during a birth peak and was associated with a slight increase in the mortality rate with no significant statistical significance. The colony comprises higher percentages of immunocompromised animals during birth peaks, namely females in late pregnancy and juveniles. Moreover, higher animal densities lead to increased stress levels, which are known to increase the likelihood of intra- and interspecies transmission of viral infections [2]. It is likely that the same applies for bacterial infections. Overall, *Y. pseudotuberculosis* was isolated from three bats of the 112 examined animals (2.67%), two of which displayed severe histopathological lesions (ID1 and 2). Interestingly, ID1 was a female that had aborted approximately 1 week prior to death*.* This finding may indicate that *Y. pseudotuberculosis* may cause abortion in this bats species as it does in ewes and goats [12]. In addition, three of the examined bats (ID10, 12 and 13) displayed a mild to moderate interstitial pneumonia, but no *Y. pseudotuberculosis*, nor any other bacteria were isolated *postmortem* from these animals.

Ingestion of food contaminated with the feces of *Yersinia* reservoir animals such as wild rats and birds (pigeons, crows) represents a likely source of infection in zoological collections [13, 14]. However, similarly to other studies reporting outbreaks of yersiniosis in zoological collections, the source of *Y. pseudotuberculosis* infection in this case was not identified. Although this colony was kept in an indoor-housed dome, rodents and also birds, namely house sparrows (*Passer domesticus*), occasionally enter the facility. In addition, due to a rigorous health screening protocol and quarantine, it is unlikely that the cohabitant nocturnal species may have introduced the pathogens.

In the outbreaks previously described in captive colonies of *R. aegyptiacus,* 20–70% of the animals exhibited gross evidence of *Y. pseudotuberculosis* infection [1, 8]. In our case, the infection rate in *C. perspicillata* was rather low and there was no statistically significant increase in the mortality rate. This may suggest a lower disease susceptibility of *C. perspicillata* compared to *R. aegyptiacus* to *Y. pseudotuberculosis* infection*.* Alternatively, a variant outcome might be related to the expression of different virulence factors. One of the *R. aegyptiacus* outbreaks described in the literature was caused by an infection with *Y. pseudotuberculosis* serotype 4b expressing *virF* and *inv* genes, which are associated with increased virulence and invasiveness, respectively [8]. In addition, this strain harbored the superantigenic toxin YPMa, which is known to lead to acute systemic infection in humans [8]. The strain causing the other described outbreak in *R. aegyptiacus* was not further characterized [1]. In our case, we found differences between the isolates of the two sick animals (16–1261/1, 16–1261/2) and of the clinically healthy female gravid bat (16–457/29) concerning the virulence genes. In all three positive bats we isolated Y. *pseudotuberculosis* serotype O:1 and biotype 1 belonging to the sequence type ST90 and expressing the *ail* gene, but detection of the *virF* and *YadA* genes was restricted to isolate 16–457/29 from the clinically healthy bat. This finding indicates that subclinical *Yersinia* infections may occur in *C. perspicillata* comparably to what is described in rodents and birds [2].

Culling ¼ of the population seems to have been sufficient to control the spread of *Y. pseudotuberculosis* within this colony without subsequent culling or antibiotic treatment, possibly due to a reduction of stress levels. Rigorous rodent and bird control measures within the zoo area are the most appropriate prophylactic approach to prevent further cases of *Yersinia* infection. It is known that *Y. pseudotuberculosis* can survive and replicate in the soil and in aquatic environments outside of hosts for months to years [15], namely in association with entomopathogenic nematodes [16] or *Acanthamoeba castellanii* trophozoites [17]. However, it has been suggested that the reinfection risk is likely to strongly decrease following 9 months of persistence in the soil since *Y. pseudotuberculosis* loses its virulence genes following adaptation to a saprophytic lifestyle [18]. In fact, no further cases of *Yersinia* infection were identified in the *C. perspicillata* colony, in the cohabitant zoological species and in the animal caretakers since this event described here took place.

## Conclusion

This report describes the occurrence of *Y. pseudotuberculosis* infections in an indoor-housed colony of *C. perspicillata*, which could be stopped by culling of ¼ of the colony. The above-described findings show that *C. perspicillata* are susceptible to infection with *Y. pseudotuberculosis* leading to lethal yersiniosis, but they don’t seem to represent a significant *Y. pseudotuberculosis* reservoir. Moreover, the infection risk for cohabitant nocturnal animals and the zoonotic potential seems to be low since no *Yersinia* infection has been diagnosed since April 2016 in this zoo.

## Material and methods

This study did not require approval by an institutional and/or licensing committee since it was not an experimental study, but part of a clinical and pathological veterinary diagnostic case that occurred in a zoo setting. All described methods were carried out in accordance with relevant guidelines and regulations, including the ARRIVE guidelines for in vivo research [19] and the ​guidelines for the euthanasia of animals from the American Veterinary Medical Association [20].

### Animals

**Fig. 2:**
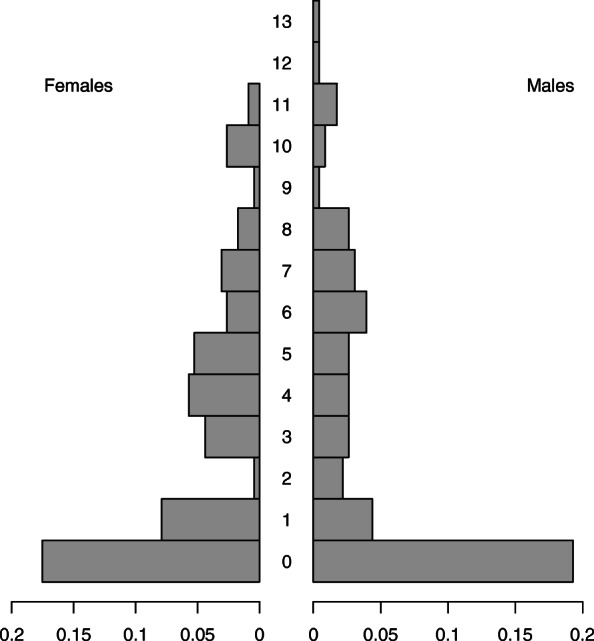
Age structure of the colony of captive *C. perspicillata* based on data from 2015. The age in years and the proportion of individuals within the population are represented on the y and x axes, respectively

At the time of the outbreak, the affected *C. perspicillata* colony comprised approximately 400 individuals of varying ages (Fig. 2). These animals were kept in a 40 m-diameter dome with an artificial cave for roosting and fed 3 times a day ad libitum. The bats also have access to the food plates of the cohabitant nocturnal animals. Food availability, temperature, and diurnal rhythm remained constant throughout the year. Due to the stable environmental conditions, birth events were spread over the entire year, with relative female reproductive synchronization. Inter-zoo exchanges and occasional population culling controlled population growth. Several other nocturnal animal species were present in the same dome, namely Brazilian porcupines (*Coendou prehensilis*), Lowland pacas (*Cuniculus paca*), night monkeys (*Aotus lemurinus griseimembra*), ocelots (*Leopardus pardalis*), and crab-eating raccoons (*Procyon cancrivorus*). The number of visitors walking through the cave varied significantly depending on seasonality and weather conditions. The health status of all animals present in the dome was continuously monitored, and all dead animals were routinely submitted for necropsy, histopathological and microbiological examination. All the bats that were culled for colony depopulation purposes following *Y. pseudotuberculosis* diagnosis within the colony were individually trapped, anesthetized with isoflurane (Nicholas Piramal I, Mumbai, India) using a rodent nosecone non-rebreathing system (Rothacher Medical, Heitenried, Switzerland), and humanly killed by cervical dislocation, in accordance with previously described protocols [21, 22].

### Pathological examination

All necropsy and histopathological examinations carried out within the scope of this study were performed at the Institute for Animal Pathology, University of Bern, Switzerland. Each examined animal was weighed, and the reproductive status was recorded. A full necropsy was performed of each animal, and liver, lung, spleen and intestine were sampled for bacteriological analysis. Necropsy instruments were cleaned with 70% ethanol before and after each organ sampling to prevent cross contamination. All internal organs (liver, spleen pancreas, adrenals, reproductive tract, urinary bladder, trachea, lung, heart, thymus, salivary glands, lymph nodes, brain, skeletal muscle, skin, bone marrow, and gastro-intestinal tract) were sampled and immediately fixed in 4% neutral buffered formalin, embedded in paraffin, cut at 4 μm, and stained with haematoxylin and eosin (HE) according to routine laboratory procedures. Histopathological interpretation was performed by certified veterinary pathologists for all embedded organs.

### Statistic evaluation of the mortality rate

An autoregressive model based on the monthly mortality recorded between January 2014 and April 2016 by the private zoo was used to analyse fluctuations in mortality rate. The “arima” function (order: 1,0,0) from R (version 3.2.3) was used to calculate the residuals and the function “locate.outliers” (package “tsouliers”). The critical value to consider t-values significant was set at 3.5 [23].

### Microbiological examination

Bacterial cultures from the first 13 bats that died between February and April 2016 were performed immediately after necropsy at the Institute of Veterinary Bacteriology, University of Bern, Switzerland. A loopful of material for each organ was streaked on BD Trypticase Soy Agar with 5% Sheep Blood (TSA SB, Becton Dickinson and on Brolac Agar (Thermo Fisher Diagnostics AG, Pratteln Switzerland) or MacConkey agar (Thermo Fisher Diagnostics AG, Pratteln Switzerland) for the lungs. Plates were incubated aerobically at 37 °C up to 48 h. TSA SB plates ware incubated in a 5% CO_2_ atmosphere. Species identification was done by matrix-assisted laser desorption/ionisation time-of-flight mass spectrometry (MALDI TOF MS, Bruker Daltonics GmbH, Bremen, Germany). Bacterial cultures from the 99 culled bats were performed immediately after necropsy at the Institute for Food Safety and Hygiene, Section of Veterinary Bacteriology, University of Zurich, Switzerland. The collected samples from liver, lung, and spleen were pooled and mashed via pestle homogenisation. A loopful of this mixture and the intestine were directly streaked onto a selective *Yersinia* agar (CIN agar; Thermo Fisher Diagnostics AG, Pratteln Switzerland) and incubated for approximately 48 h at 30 ± 1 °C. Additionally, a loopful of the mixture was inoculated in an enrichment-broth (EE-broth, Mossel; Thermo Fisher Diagnostics AG, Pratteln Switzerland), incubated overnight at 30 ± 1 °C, streaked onto CIN agar and again incubated overnight at 30 ± 1 °C. Suspicious colonies from the CIN agar were picked and tested for urease activity (Urea broth, Christensen and Maslen; Thermo Fisher Diagnostics AG, Pratteln Switzerland), which was incubated overnight at 30 ± 1 °C. If the urea broth was positive, subcultures were done onto Columbia blood agar containing sheep blood (Thermo Fisher Diagnostics AG, Pratteln Switzerland) at 30 ± 1 °C. Species identification was done by biochemical test using the automated VITEK® 2 Compact system (bioMérieux, Marcy l’Etoile, France).

In order to determine the serotype, biotype, sequence type and the expression of virulence genes *virF*, *yadA* and *ail*, the three isolates were further characterized at the Department of Food Hygiene and Environmental Health, Faculty of Veterinary Medicine, University of Helsinki, Finland. Serotyping of the isolates was performed by slide agglutination with commercial antisera O:1 - O:6 (Denka Seikan, Tokyo, Japan). The whole-genome sequencing was performed according to Joutsen et al. [24]. The sequence type was assigned using the multilocus sequence typing database described by Hall et al. [25]. The *virF* and *yadA* genes on the virulence plasmid and the chromosomal *ail* gene were detected by PCR according to Joutsen et al. [26].

## Data Availability

All data generated or analyzed during this study are included in this published article.

## Abbreviations

PCR: Polymerase chain reaction
HE: Haematoxylin and eosin
